# German translation, cultural adaptation and validation of the Person-Centred Practice Inventory—Staff (PCPI-S)

**DOI:** 10.1186/s12913-023-09483-8

**Published:** 2023-05-09

**Authors:** Christoph von Dach, Nanja Schlup, Stefan Gschwenter, Brendan McCormack

**Affiliations:** 1 Bern University of Applied Science, Bern, Switzerland; 2 Solothurn’s Hospital Group, Solothurn, Switzerland; 3 Queen Margaret University, Edinburgh, UK; 4 University Hospital of Psychiatry, Bern, Switzerland; 5 Karl Landsteiner University of Health Sciences, Krems, Austria; 6 Sydney Nursing School, The University of Sydney, Sydney, Australia

**Keywords:** Acute care, Cultural adaption, Factor analysis, Health care professionals, Instrument, Person-centredness, Psychometric analysis, Translation

## Abstract

**Background:**

The person-centred practice framework represents the cornerstone of a middle-range theory. Internationally, person-centredness has become an increasingly common topic. The measurement of the existence of a person-centred culture is complex and subtle. The Person-Centred Practice Inventory—Staff (PCPI-S) measures clinicians’ experience of a person-centred culture in their practice. The PCPI-S was developed in English. Therefore, the aims of this study were (1) to translate the PCPI-S into German and to cross-culturally adapt and test in the acute care setting (PCPI-S aG Swiss) and (2) to investigate the psychometric properties of the PCPI-S aG Swiss.

**Methods:**

The two-phase investigation of this cross-sectional observational study followed the guidelines and principles of good practice for the process of translation and cross-cultural adaptation of self-reporting measures. Phase 1 involved an eight-step translation and cultural adaptation of the PCPI-S testing in an acute care setting. In Phase 2, psychometric retesting and statistical analysis based on a quantitative cross-sectional survey were undertaken. To evaluate the construct validity, a confirmatory factor analysis was implemented. Cronbach’s alpha was used to determine the internal consistency.

**Results:**

A sample of 711 nurses working in a Swiss acute care setting participated in testing the PCPI-S aG Swiss. Confirmatory factor analysis indicated a good overall model fit, validating the strong theoretical framework, which underpins the PCPI-S aG Swiss. Cronbach’s alpha scores demonstrated excellent internal consistency.

**Conclusion:**

The chosen procedure ensured cultural adaptation to the German-speaking part of Switzerland. The psychometric results were good to excellent and comparable with other translations of the instrument.

## Background

Person-centred practice (PCP) is “an approach to practice established through the formation and fostering of healthful relationships between all care providers, service users and others significant to them in their lives. It is underpinned by the values of respect for persons, individuals’ right to self-determination, mutual respect and understanding. It is enabled by cultures of empowerment that foster continuous approaches to practice development” [1(p3)].

The PCP Framework represents the cornerstone of the middle-range theory of person-centred practice. The main result is defined as a healthful culture, which includes a good care experience, the involvement of the patient and the caregiver in care and a feeling of well-being for all persons involved. The recognition and influence of person-centred health care are increasing and may serve as a remedy to the current continuing crisis in health care worldwide [1].

Internationally, person-centredness has become an increasingly common topic in health care and in everyday health care discussions [2]. Evidence on a global scale shows the benefits of a person-centred approach to health care delivery at all systems levels [3]. PCP focuses on a holistic perspective of the person and avoids the reduction of the person to an “illness” [4]. Furthermore, it is based on the person’s world of daily experience and individual will, preferences, values and beliefs [5]. This leads to fundamental advantages, such as shorter hospital stays, the preservation of functional abilities and an increased health-related quality of life [6]. Furthermore, research has generated insights into the cultural as well as contextual challenges associated with the implementation of person-centred practices [7-11]. The theory of person-centred practice framework [1] leads to a paradigm shift from one-person medicine, where the focus is on the patient, to two person-medicine, which integrates the physician, the nurse and all the other medical staff as the persons in focus [12]. A cornerstone for establishing PCP is the internationally recognised theoretical PCP Framework, developed by McCormack & McCance [1]. It supports health care teams and individual professionals in health care to understand the dimensions of person-centredness and how these dimensions may be operationalised in clinical practice. As such, it helps practitioners understand the impact of their practice [13]. The PCP Framework contains four constructs*: prerequisites, care environment, person-centred processes and person-centred outcomes.* The focus of these four constructs has been contextualised through the perspective of the macro health care context. The four constructs are positioned as the preconditions for the attributes of staff, the health care organisation and the environment, which have been prepared in order to support the development of a healthful culture as the main goal of PCP. The measurement of a person-centred culture is complex and subtle [14]. For this reason, approaches used to measure and evaluate a person-centred culture and its components have to be holistic and focus on the different persons who are part of the culture. Furthermore, an evaluation has to consider different components and research approaches. The three most commonly used research methods to measure person-centred culture are surveys and interviews with people using health services, surveys of clinicians and the observation of clinical encounters [15]. However, currently several instruments exist to measure person-centredness, mostly proxy measures and focused on patient-centred outcomes. Only a few instruments have been developed that measure person-centred culture [13]. One instrument used to measure clinicians’ experience of person-centred culture in their individual clinical practice is the Person-Centred Practice Inventory—Staff (PCPI-S). The PCPI-S has been translated into several languages, including Norwegian, Swedish, Malay, Portuguese, Slovenian and Dutch. The first of these translations was into the Norwegian language [16]. The original PCPI-S was developed for different settings, including acute care [16]. The German translation reported in this paper came about because of the need for an instrument to measure person-centred culture in an acute care setting. At the starting point of the translation project, no other German translation was available. Later, a German translation was carried out in Austria [17]. This translation was done in order to evaluate the effects of implementing a framework for person-centred culture in long-term care settings in Austria [17].

## Methods

### Aims

The aims of this study were (1) to translate the PCPI-S into German and to adapt this translation cross-culturally to the acute care setting (PCPI-S aG Swiss); and (2) to investigate the psychometric properties of the PCPI-S aG Swiss.

### Study design and setting

This was a cross-sectional observational study conducted at a large hospital group in Switzerland, the operator of the public hospitals in the canton of Solothurn. The Solothurn’s hospital group (SoH) consists of four acute care hospitals, providing primary and mental health services for more than 30,000 adult inpatients and about 180,000 adult outpatients a year and with around 4,000 health care professionals employed.

### Instrument

The PCPI-S contains 59 items that assess 17 different constructs aligned to the PCP Framework: professionally competent; developed interpersonal skills; being committed to the job; knowing self; clarity of beliefs and values; skill mix; shared decision-making systems; effective staff relationships; power sharing; potential for innovation and risk-taking; the physical environment; supportive organisational systems; working with patients’ beliefs and values; shared decision-making; engagement; having a sympathetic presence; and providing holistic care. All of which are contained within the three main constructs of prerequisites, care environment and person-centred processes, with person-centred outcomes not being measured by the instrument.

Items are measured on a 5-point Likert-type scale (1 = “strongly disagree” to 5 = “strongly agree”). This was recoded as ranging from 0 to 4 (“strongly disagree” to “strongly agree”, previously 1–5). Mean scores on the 5-point Likert scale ranged from 2.0 to 3.6, with 0 representing the most negative and 4 the most positive answer. Calculating mean factor scores of the items aligned with the construct allows for scoring of the construct [18]. This is achieved by summing up each respondent’s item scores within a construct, divided by the number of items and transforming it into a scale ranging from 0–100, with higher scores representing higher levels of agreement. The PCPI-S also includes socio-demographic information on gender, education and years of professional experience [14].

Constructs and items of the original PCPI-S were determined based on theory and through an iterative Delphi study. The instrument was subsequently tested in a sample of 703 nurses working in different acute care hospital settings across Ireland (adult services; children and young people; primary care and older people; mental health and learning disability). Fit indices of PCPI-S were the chi-squared test of model fit value 4517, degrees of freedom 1516, P-value < 0.001; root mean square error of approximation (RMSEA) 0.053; 90% RMSEA 0.051–0.055; comparative fit index (CFI) 0.951 [14].

#### Phase 1: translation and cultural adaptation

**Fig. 1:**
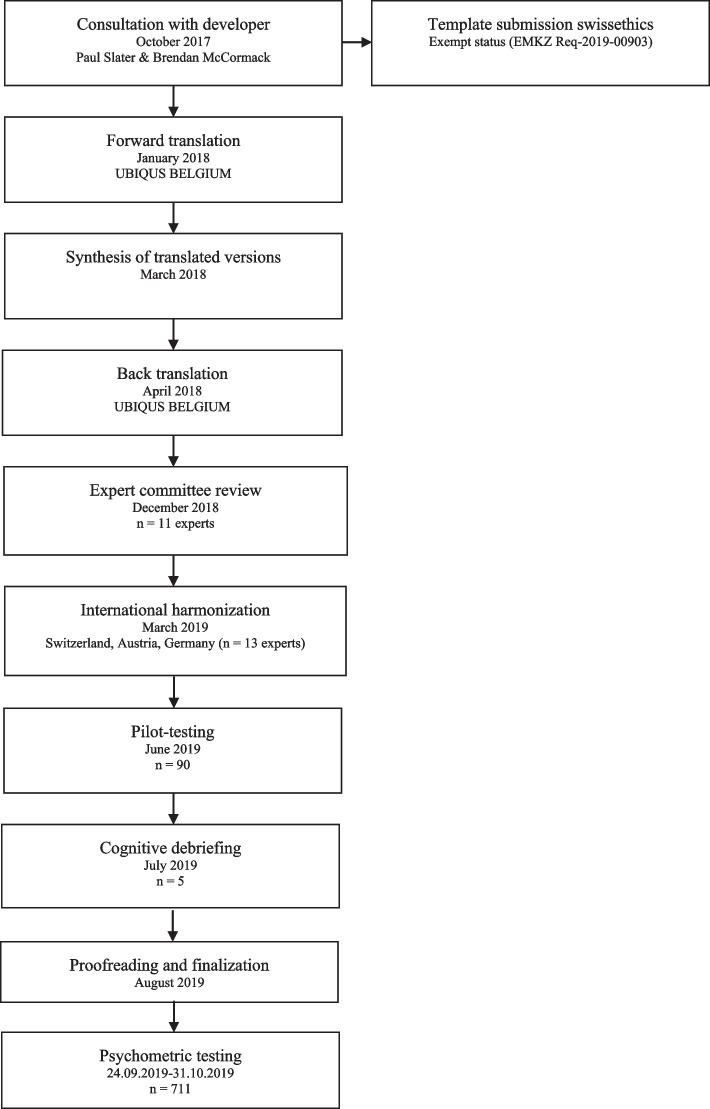
Steps of Translation

The investigation followed the guidelines for the process of cross-cultural adaptation of self-reporting measures [19] and the principles of good practice for the translation and cultural adaptation process of the International Society for Pharmacoeconomics and Outcomes Research (ISPOR) task force for translation and cultural adaptation [20]. The developers (Slater, McCance and McCormack) granted permission to translate and culturally adapt the PCPI-S. All comments, rationales and decisions during the process were recorded in writing and stored by the study team. All steps of translation and cultural adaption were summarised in Fig. 1 as a flow diagram.

##### Step 1: forward translation

The forward translation was conducted in January 2018 by two independent translators of a professional and certified translation service [21]. The translators were not familiar with the PCP concept, however they were both professional translators. The translations (T1 + T2) included translation of the PCPI-S instructions, the socio-demographics and the item content and response options from English to German.

##### Step 2: synthesis

For synthesis, T1 and T2 were first reconciled through a single forward translation (T-12a) comparing the original version of PCPI-S. The project leader did this in March 2018. Secondly, an expert panel was conducted to 1) compare T-12a against PCPI-S to identify possible discrepancies and 2) develop a T-12b version, considering T-12a, the original PCPI-S and cultural or linguistic discrepancies. The expert panel consisted of a convenient sample of 13 nursing experts with master’s or bachelor’s degrees working in acute care at one of the hospitals of the hospital group and took place in April 2018.

##### Step 3: back translation and synthesis

The translation of T-12b back to English was conducted in April 2018 by two independent translators [21]. The translators were not familiar with the forward translation versions nor with the PCP concept and access to the original English version of the PCPI-S was not permitted. The back translations (BT1 + BT2) included the PCPI-S instructions, the socio-demographics and the item content and response options. Considering PCPI-S, T-12b, BT1 and BT2, the first German version of PCPI-S aG Swiss was developed in April 2018 by two clinical nurse specialists, one with a doctorate (Doctor of Nursing Practice), the other with a master’s degree in nursing.

##### Step 4: expert committee review

In December 2018, an expert committee review was conducted to ensure cross-cultural adaptation of the newly developed PCPI-S aG Swiss for the acute care setting. Thus, conceptual, methodological and linguistic experts, as well as nurses representing the target population, were invited to participate in the review. In total, 10 Swiss experts, excluding the investigator, participated in the review: two conceptual and methodological experts with a master’s degree in nursing (familiar with PCP and guidelines on translation and cultural adaption); six nurses of different educational levels (one ward manager, two licensed practical nurses, two nurses with a bachelor’s degree, and one advanced practice nurse with a master’s degree); and two language experts (native English and German speakers, not familiar with the concept). Participating nurses’ native language was German, with intermediate or advanced levels of English.

The expert committee compared the PCPI-S aG Swiss and T-12b against the original PCPI-S to identify possible discrepancies. Furthermore, semantic, idiomatic, experiential and conceptual equivalence were considered. By reaching consensus on the translations of the instructions, the socio-demographics and the item content and response options, the pre-final version of the PCPI-S aG Swiss was developed.

##### Step 5: international harmonisation

The PCP Framework was translated into German and culturally adapted to Swiss, German and Austrian care settings consecutively. Thus, overlapping terms of both the PCP Framework and the PCPI-S aG Swiss were adjusted in a consensus meeting conducted by the lead investigator at in March 2019. In total, 12 experts, excluding the lead investigator, participated in a consensus meeting: nine Swiss (conceptual, methodological and linguistic), one Austrian (conceptual and methodological), and two German (conceptual and methodological) experts participated in that meeting. Three participants held doctorates, five had master’s degrees and three held bachelor’s degrees in nursing. One person had a master’s degree in linguistics. McCance and McCormack participated in the meeting online and were responsible for clarifying uncertainties in meaning/understanding. Necessary adjustments arising from the consensus meeting were adapted to the PCPI-S aG Swiss by the study team in March 2019.

##### Step 6: pilot testing

To evaluate the quality and readability of the PCPI-S aG Swiss in the target population, the PCPI-S aG Swiss was pilot tested in June 2019. The study team conveniently selected two units per hospital. All nurses working on these units were invited to complete the PCPI-S aG Swiss and to identify words and phrases that were difficult to understand. The inclusion criteria for the nurses were: working in direct care on the unit, a good understanding of German and the willingness to complete the questionnaire. Trainee nurses were excluded. The PCPI-S aG Swiss was sent as a paper-based questionnaire from the second author to the unit leaders via the internal postal system and then distributed by the unit leaders to nurses. The survey data were analysed by the first and second authors.

##### Step 7: cognitive debriefing

Concomitant with pilot testing, the PCPI-S aG Swiss was verified for cognitive equivalence in July 2019. To achieve this, five semi-structured interviews with a convenient sample of nurses working across the four SoH hospitals were conducted by the first and second authors. The participants were asked to repeat each item in their own words and identify any words or phrases that were difficult to understand.

##### Step: 8: proofreading and finalising

Considering comments from the pilot testing and the cognitive debriefing, the final adjustments of the PCPI-S aG Swiss were carried out by the first and second authors in August 2019. For completion, the questionnaire was also checked for any remaining spelling, diacritical (special characters), grammatical or other formal errors.

#### Phase 2: psychometric testing of PCPI-S aG Swiss

For psychometric testing, all nurses working on inpatient units across the four hospitals were invited to complete the PCPI-S aG Swiss during a four-week period in October 2019. Trainee nurses were excluded. To this end, a link to the PCPI-S aG Swiss online questionnaire was sent via internal email addresses, with all items set as mandatory. A reminder was sent via internal email addresses after two weeks. The inclusion criteria for the nurses were: working in direct acute care, a good understanding of German and the willingness to complete the questionnaire.

Psychometric analysis was performed using RStudio version 1.2.5001, with lavaan package version 0.6–5 [22] for structural equation modelling. In order to examine the theoretical measurement model, confirmatory factor analysis (CFA) was utilised. With reference to the PCP Framework, the three main constructs of the questionnaire (prerequisites, care environment, person-centred processes) were modelled and analysed independently of each other, as each represents a separate construct of the PCP Framework.

Similar to the original testing of the PCPI-S [14], several items show skewness and kurtosis, suggesting non-normality of the data. Therefore, the maximum likelihood with robust standard errors (MLR) estimators was used for CFA.

Factor loadings greater than 0.3 were accepted. Regarding model fit, acceptable statistics were set at RMSEA of 0.08 or below; a 90% higher bracket below 0.09; a CFI of 0.90 or higher; and a standardised root mean square residual (SRMR) lower than 0.10, indicating a good model fit [23].

Additionally, Cronbach’s alpha was calculated to analyse the internal consistency of the instrument, with values of at least 0.7 to be considered acceptable, good between 0.8–0.9 and excellent above 0.9 [23].

## Results

### Phase 1: translation and cultural adaptation

*The synthesis of the forward translations* (T1 + T2) to the T-12a version included revision of 47 of 59 items, either due to grammatical or linguistic need. Translations regarding the meanings of “opportunities”, “demonstrate respect”, “evidence”, “ways of being” and “skill mix” were discussed, and the translations were changed. *The expert panel synthesis* to the T-12b version consisted of a revision of 18 of 59 items due to grammatical or linguistic need. Translations regarding the meanings of “task”, “care experience”, “ways of being” and “things” were discussed, and the translations were revised. Item 23 was expanded with examples of decision-making forums.

*Synthesis after back translation* only included changes due to grammatical or linguistic need in four of 59 items. Translations regarding the meanings of “extent” and “participation” were discussed, and the translations were revised.

*In the expert committee review,* the socio-demographic item of gender was expanded with “other”, and an item to assess the participant’s age was added. The translation of “opportunities” was revised and an explanation of “evidence” was created in a footnote. This was because the term “opportunities” did not support better understanding and “evidence” was explained as a holistic term composed of the four dimensions of literature, expert knowledge, patient experience and care environment. The terms “care”, “caring” and “care process” were discussed in detail and needed further inquiry by the developers. In the German language, the term “care” is attributed to the nursing profession, but in the English language, caring has a more comprehensive meaning and may include other health professions. The first author clarified this uncertainty after an expert committee review with McCormack—all health professionals were intended, and the focus was not only on nursing professionals.

*International harmonisation* included discussion about the meaning of “care” and “caring” as well. The consensus reached was that all professions should be included in the concept of PCP. Furthermore, the translation of “person-centredness” was discussed, and the translation changed from “Personenzentrierung” to “Personzentrierung”. The meaning of care and the newly created translation of “person-centredness” were revised in the PCPI-S aG Swiss after the consensus meeting by the first and second authors.

*Pilot testing* included the data of 106 returned PCPI-S aG Swiss paper questionnaires, with an overall response rate of 63%. Most of the participants were registered nurses (70%), 24% were licensed practical nurses, 3% nursing aides, and 3% others. 87% were female, 12% male and 1% other. Almost half of the participants had over 10 years of professional experience (48%). In total, 27 feedback responses concerning 16 items were returned: 11 unanswered questions, eight comments and eight markings of words or parts of sentences. Three items were revised after pilot testing due to more than two feedback responses per item, respectively, due to linguistic need.

*Cognitive debriefing* was done by interviewing five female registered nurses working in an acute care setting, with ages ranging between 19 and 59 years. These nurses had two to 12 years of professional experience in nursing, working either at an internal medicine or surgery unit. Overall, the interview participants understood the questions. There were eight words that were not fully clear. The result was discussed within the research team and minor changes were made.

### Phase 2: psychometric testing of PCPI-S aG Swiss

In total, 1,130 nurses received the link to complete the PCPI-S aG Swiss. 834 surveys were returned, of which 123 were blanks, leaving *n *= 711 surveys without missing data for analysis. This resulted in a response rate of 63%, comfortably exceeding the respondent-to-item ratio necessary to perform CFA for each construct [24]. Answering the questionnaire took an average of 6.3 min. The time was automatically measured by the electronic tool used for the questionnaire. The nurses who participated worked on 55 units across the four SoH hospitals, while 90% of these worked in a somatic setting and 10% in a psychiatric acute care setting.

As represented in Table 1, 90% of the participants were female, with the largest age groups being represented by 19–29-year-olds (30%) and 30–39-year-olds (25%). Most participants were registered nurses (61%) or licensed practical nurses (17%). More than half (54%) of the participants had over 10 years of professional experience.

**Table 1:** Socio-demographic sample characteristics (*N* = 711)

Value	N (%)
Setting	
Somatic	637 (90)
Psychiatric	74 (10)
Gender	
Female	641 (90)
Male	68 (10)
Other	2 (0)
Age	
≤ 18 years	40 (6)
19–29 years	211 (30)
30–39 years	180 (25)
40–49 years	117 (16)
50–59 years	126 (18)
≥ 60 years	37 (5)
Highest work-related education	
Master’s degree	21 (3)
Registered nurses	432 (61)
Licensed practical nurses	120 (17)
Nursing aides	9 (1)
Medical office assistant	5 (1)
Others	124 (17)
Professional experience	
< 1 year	60 (8)
1–5 years	165 (23)
6–10 years	104 (15)
> 10 years	382 (54)

*N* = Sample size, % = Percentage

Overall, all items were rated positively. Mean scores on a 5-point Likert scale ranged from 2.0 to 3.6, with 0 representing the most negative and 4 the most positive answer.

Mean factor scores were 79 for the construct *prerequisites*, 69 for *care environment* and 77 for *person-centred processes*.

As represented in Table 2, Cronbach’s alpha scores were good for the constructs *prerequisites* and *care environment*, with scores of 0.865 and 0.897 respectively, and excellent for the construct *person-centred processes*, with a score of 0.906*.*

**Table 2:** Mean scores and factor loadings of the PCPI-S aG

Construct scores and items	Mean	SD	Factor loading	SE
**Prerequisites (Cronbach’s alpha)**				0.865
Professionally competent				
I have the necessary skills to negotiate care options	3.27	0.63	0.505	0.042
When I provide care, I pay attention to more than the immediate physical task	3.50	0.60	0.522	0.048
I actively seek opportunities to extend my professional competence	3.30	0.61	0.530	0.032
Developed interpersonal skills				
I ensure I hear and acknowledge others’ perspectives	3.36	0.58	0.586	0.034
In my communication, I demonstrate respect for others	3.60	0.56	0.575	0.038
I use different communication techniques to find mutually agreed solutions	3.17	0.63	0.660	0.028
I pay attention to how my non-verbal cues impact my engagement with others	3.06	0.65	0.622	0.031
Being committed to the job				
I strive to deliver high-quality care to people	3.56	0.56	0.543	0.030
I seek opportunities to get to know the person and their family in order to provide holistic care	2.98	0.75	0.640	0.030
I go out of my way to spend time with people receiving care	3.20	0.68	0.628	0.033
I strive to deliver high-quality care that is informed by evidence	3.04	0.65	0.569	0.033
I continuously look for opportunities to improve the care experience	3.07	0.63	0.652	0.028
Knowing self				
I take time to explore why I react as I do in certain situations	2.99	0.67	0.639	0.034
I use reflection to check out if my actions are consistent with my ways of being	3.18	0.64	0.733	0.035
I pay attention to how my life experiences influence my practice	3.09	0.71	0.564	0.039
Clarity of beliefs and values				
I actively seek feedback from others about my practice	2.88	0.79	0.473	0.039
I challenge colleagues when their practice is inconsistent with our team’s shared values and beliefs	2.75	0.78	0.751	0.028
I support colleagues to develop their practice to reflect the team’s shared values and beliefs	2.91	0.69	0.704	0.031
**The Care Environment (Cronbach’s alpha)**				0.897
Skill Mix				
I recognise when there is a deficit in knowledge and skills in the team and its impact on care delivery	2.71	0.89	0.444	0.069
I am able to make the case when the skill mix falls below acceptable levels	2.58	0.82	0.678	0.071
I value the input from all team members and their contributions to care	3.23	0.66	0.467	0.069
Shared decision-making systems				
I actively participate in team meetings to inform my decision making	3.15	0.80	0.520	0.043
I participate in organisation-wide decision-making forums that impact on practice	2.36	0.99	0.595	0.037
I am able to access opportunities to actively participate in influencing decisions in my directorate/division	2.02	1.06	0.669	0.036
My opinion is sought in clinical decision-making forums (e.g., ward rounds, case conferences and discharge planning)	2.56	0.95	0.520	0.039
Effective staff relationships				
I work in a team that values my contribution to person-centred care	2.94	0.70	0.757	0.029
I work in a team that encourages everyone’s contribution to person-centred care	2.78	0.74	0.820	0.021
My colleagues positively role model the development of effective relationships	2.68	0.79	0.690	0.030
Power sharing				
The contribution of colleagues is recognised and acknowledged	2.85	0.73	0.643	0.032
I actively contribute to the development of shared goals	3.08	0.62	0.562	0.038
The leader facilitates participation	2.88	0.81	0.751	0.025
I am encouraged and supported to lead developments in practice	2.88	0.76	0.776	0.023
Potential for innovation and risk taking				
I am supported to do things differently to improve my practice	2.79	0.80	0.697	0.038
I am able to balance the use of evidence with taking risks	2.93	0.76	0.326	0.052
I am committed to enhancing care by challenging practice	3.02	0.63	0.394	0.056
The physical environment				
I pay attention to the impact of the physical environment on people’s dignity	3.47	0.60	0.403	0.040
I challenge others to consider how different elements of the physical environment impact person-centredness (e.g., noise, light and heat)	2.94	0.73	0.716	0.038
I seek out creative ways of improving the pep environment	2.62	0.83	0.659	0.037
Supportive organisational systems				
In my team, we take time to celebrate our achievements	2.27	0.88	0.641	0.029
My organisation recognises and rewards success	2.30	0.83	0.717	0.026
I am recognised for the contribution that I make to people having a good experience of care	2.57	0.78	0.752	0.026
I am supported to express concerns about an aspect of care	2.47	0.76	0.752	0.028
I have the opportunity to discuss my practice and professional development on a regular basis	2.73	0.77	0.540	0.035
**Person-centred Processes (Cronbach’s alpha)**				0.906
Working with patients’ beliefs and values				
I integrate my knowledge of the person into care delivery	2.93	0.63	0.586	0.032
I work with the person within the context of their family and carers	2.86	0.67	0.706	0.030
I seek feedback on how people make sense of their care experience	2.73	0.74	0.716	0.034
I encourage people to discuss what is important to them	3.14	0.64	0.696	0.027
Shared decision-making				
I include the family in care decisions where appropriate and/or in line with the person’s wishes	2.95	0.75	0.718	0.028
I work with the person to set health goals for their future	2.83	0.79	0.699	0.035
I enable people receiving care to seek information about their care from other healthcare professionals	2.75	0.75	0.645	0.034
Engagement				
I try to understand the person’s perspective	3.34	0.57	0.671	0.034
I seek to resolve issues when my goals for the person differ from their perspectives	3.14	0.55	0.740	0.032
I engage people in care processes where appropriate	2.97	0.64	0.675	0.035
Having a sympathetic presence				
I actively listen to people receiving care to identify unmet needs	3.42	0.60	0.710	0.028
I gather additional information to help me support people receiving care	3.17	0.67	0.743	0.024
I ensure my full attention is focused on the person when I am with them	3.39	0.60	0.708	0.030
Providing holistic care				
I strive to gain a sense of the whole person	3.45	0.58	0.789	0.025
I assess the needs of the person, taking account of all aspects of their lives	3.13	0.64	0.732	0.029
I deliver care that takes account of the whole person	3.19	0.62	0.741	0.029
**PCPI-S aG (Cronbach’s alpha)**				0.946

*SD* standard deviation, *SE* standard error

Factor loadings ranged from 0.44 to 0.82, and all loadings were statistically significant (P < 0.05), indicating a valid contribution to the model. The model fit indices across all three constructs demonstrate a psychometrically sound instrument, with RMSEA ranging from 0.049 to 0.077, 90% RMSEA ranging from 0.07 to 0.083 for all constructs and a CFI between 0.83 and 0.93, with only the construct *care environment* being slightly below the desired cut-off value, and SRMR ranging from 0.042 to 0.074, indicating good model fit. Detailed scores for the model fit are presented in Table 3.

**Table 3:** Fit Statistics of PCPI-S aG

Constructs	RMSEA	90% RMSEA	CFI	SRMR
Prerequisites	0.049	0.043–0.055	0.932	0.042
The Care Environment	0.077	0.073–0.081	0.830	0.074
Person-centred Processes	0.077	0.070–0.083	0.917	0.048

*RMSEA* Root mean square error of approximation, *CFI* Comparative fit index, *SRMR* Standardised root mean square residual

## Discussion

The internal consistency for the PCPI-S aG Swiss achieved similar values to the Austrian and Norwegian translations [16, 17]. The model fit was positive overall, for all constructs, which is also comparable with both previously noted translations [16, 17].

Recommended as a minimum standard for applying an instrument developed in another language, investigations should include back translation and testing [25] while the Austrian translation took place with no back translation. Guidelines for the process of cross-cultural adaptation of self-reporting measures stated that linguistic equivalence requires that words and phrases reflect the same basic meaning across translations, including grammatical structures, multiple word meanings, and connotations [19]. This challenged the translation process because a European particularity is that various different countries use the German language (Austria, Germany and Switzerland).

For example while there are many similarities between the PCPI-S German Version [17] and the PCPI-S acute German Swiss (PCPI-S aG (Swiss)) reported here, they differ in the specific use of terms and the number of items.

A factor caused by the uniqueness of these countries is that they use terms and expressions differently as a result of the varying cultures of the three countries. An example is the term used for the patient in the different countries (“PatientIn”, “KundIn”, “PflegeempfängerIn”) or for the directory (“Direktion”). Furthermore, Switzerland has many German dialects, which also differ in the meanings of expressions used. There is a risk in translation of having Swiss idiosyncrasies, resulting in “Helvetisms” [26] which may refer to terms only used by German-speaking Swiss people, caused by their Swiss dialect. With this fact in mind, we provided a consensus conference between the three countries, to make the questionnaire usable all over Europe. Besides the consensus on the terms, an important effect of this process was a starting point of a shared understanding of the person-centred culture for the German-speaking part of Europe. This opened the field for the cognitive debriefing, done by five interviews with nurses in direct clinical practice. This carefully performed process ensured a profound and practical PCPI-S aG Swiss version in the German language.

The limitation may be seen in the fact that the testing was undertaken in a single hospital group of four hospitals and only by nurses. In relation to an interprofessional use of the questionnaire, it is even not possible to choose “physician”. This must be changed if it is used in an interprofessional setting. The results might have been different by having more than one institutional culture and different health care professionals participating. On the other hand, the hospital was a group with four institutions in which each had a specific culture, caused by the history of each hospital. The four hospitals were merged just over 10 years ago. Also, based on the sample size, the results are comparable with the data of the development of the instrument [14], the testing in the German language for long-term care settings [17], the cross-cultural adaptation in Malaysia [27] and the translation in Norway [16].

## Conclusions

The results presented in this paper have relevance for the further development of person-centred cultures in acute care settings in the German-speaking part of the world on two fronts. First of all, they enable insight to be gained into the person-centred culture in a team or an organisation. These results help to obtain a reference point to start or further develop person-centred cultures in acute care settings. Furthermore, they enable national and international benchmarking about the experiences of person-centred cultures among health care staff.

## Data Availability

The datasets used and/or analysed during the current study are available from the corresponding author on reasonable request. Resources and output generated by the Centre for Person-centred Practice Research, as the PCPI-S aG Swiss, can be downloaded (https://www.cpcpr.org/resources).

## Abbreviations

CFA: Confirmatory factor analysis
CFI: Comparative fit index
ISPOR: International Society for Pharmacoeconomics and Outcomes Research
MLR: Maximum likelihood with robust standard errors
PCP: Person-centred practice
PCPI-S: Person-Centred Practice Inventory—Staff
PCPI-S aG Swiss: Person-Centred Practice Inventory—Staff acute German Version
RMSEA: Root mean square error of approximation
SRMR: Standardised root mean square residual
